# Supplementary material for: Liquid Biopsy for Detection of Pancreaticobiliary Cancers by Functional Enrichment and Immunofluorescent Profiling of Circulating Tumor Cells and Their Clusters
Source: Cancers (Basel). 2024 Apr 2;16(7):1400. doi: 10.3390/cancers16071400 (PMC11010988; doi:10.3390/cancers16071400)

Supplementary Materials

Title

Liquid Biopsy for Detection of Pancreaticobiliary Cancers by Functional Enrichment and Immunofluorescent Profiling of Circulating Tumor Cells and their Clusters.

Authors

Andrew Gaya, Nitesh Rohatgi, Sewanti Limaye, Aditya Shreenivas, Ramin Ajami, Dadasaheb Akolkar, Vineet Datta, Ajay Srinivasan, Darshana Patil.

Table of Contents

Section	Title	Page
**Supplementary Methods**		
**Method S1**	Markers	**2**
**Method S2**	Antisera and Cell Lines	**2**
**Method S3**	Enrichment of CTCs	**3**
**Method S4**	Immunocytochemistry Profiling of CTCs	**3**
**Method S5**	Marker Expression in Various Cell types	**4**
**Method S6**	Marker Expression in PBC	**4**
**Method S7**	PB-CTCs in Non-malignant Conditions	**4**
**Method S8**	Analyte Stability	**4**
**Method S9**	Linearity and Limit of Detection	**5**
**Method S10**	Limit of Blank	**5**
**Method S11**	Interference	**5**
**Method S12**	Inter-Operator Agreement	**6**
**Supplementary Tables**		
**Table S1**	Marker Expression Study Cohort: PBC cases	**7**
**Table S2**	Marker Expression Study Cohort: Non-malignant Conditions	**8**
**Table S3**	Potentially Interfering Substances	**9**
**Table S4**	Inter-Operator Concordance Study: Demographics	**10**
**Table S5**	Inter-Operator Concordance Study: Findings	**11**
**Table S6**	Case Control Clinical Study: Inclusion and Exclusion Criteria	**12**
**Table S7**	Case Control Clinical Study: Participant Demographics	**13**
**Table S8**	Case Control Clinical Study: Training Set Findings	**14**
**Table S9**	Prospective Clinical Study: Participant Demographics	**15**
**Table S10**	Prospective Clinical Study: Findings	**16**
**Supplementary Figures**		
**Figure S1**	Marker Expression in Various Cell types.	**17**
**Figure S2**	PBC Stage and Marker Expression	**18**
**Figure S3**	Patient Age and Marker Expression	**19**
**Figure S4**	Patient Gender and Marker Expression	**20**

SUPPLEMENTARY METHODS

Method S1. Markers

The Test uses a multi-marker system for detection of Pancreaticobiliary Cancer associated Circulating Tumor Cells (PB-CTCs), as malignant apoptosis resistant cells expressing (positive for) Carbohydrate Antigen 19-9 (CA19.9), Mammary Serine Protease Inhibitor (Maspin), Epithelial Cell Adhesion Molecule (EpCAM) and Cytokeratins (CK), and negative for the common leucocyte antigen (CD45). CA19.9 plays a vital role in cell recognition processes and is used in the management of pancreatic, gallbladder and bile duct cancers. Maspin was originally reported to function as a tumor suppressor gene in epithelial cells, suppressing the ability of cancer cells to invade and metastasize to other tissues. Maspin protein is upregulated in pancreatic cancers but not in normal pancreatic tissue, thus aiding differentiation of pancreatic cancer from benign conditions. Maspin expression is also upregulated in gallbladder and bile duct cancers. EpCAM are membrane antigens present on epithelial cells (and carcinomas) that function in cell adhesion. CK are a family of cytoplasmic structural proteins expressed in epithelial tumors and CTCs. CD45 as a negative marker serves to differentiate CTCs from CD45 positive haematolymphoid cells. CA19.9 and Maspin are evaluated during routine diagnostic histopathological evaluation (HPE) of pancreaticobiliary tumor tissue. Co-expression of Maspin and CA19.9 is rare in organ systems outside of the Pancreas, Gallbladder and Bile Duct.

Method S2. Antisera and Cell Lines

The primary antibodies (1° Ab) used in the test include Vio B515 conjugated monoclonal recombinant human (RH) anti-CK IgG1 (Clone REA831, Miltenyi Biotech), PE-Vio 615 conjugated monoclonal RH anti-CD326 IgG1 (Clone REA764, Miltenyi Biotech), APC-Vio 770 conjugated monoclonal RH anti-CD45 IgG1 (Clone REA747, Miltenyi Biotech), unconjugated monoclonal mouse anti-Maspin IgG1 (Clone 121SLE, BioSB), and unconjugated monoclonal mouse Anti-CA19.9 IgG1 (Clone BSB-92, BioSB). The secondary antibodies (2° Ab) include Alexa Fluor™ Plus 594 conjugated polyclonal goat anti-mouse IgG (Invitrogen) for mouse anti-Maspin IgG1, and Alexa Fluor™ 594 conjugated polyclonal goat anti-mouse IgM (Invitrogen) for mouse anti-CA19.9 IgG1.The reference cell lines PL45 (human pancreatic ductal adenocarcinoma) and MOLT-3 (human acute lymphoblastic leukemia) were procured

from American Type Culture Collection (ATCC). The purity of all cell lines was confirmed by periodic Short Tandem Repeat (STR) profiling. All cell lines were also periodically tested and verified to be Mycoplasma negative.

Method S3. Enrichment of CTCs

Aliquoted blood samples (5 mL) were processed for the enrichment of circulating tumor cells (CTCs) from white blood cells (WBC) as published previously[1]. Briefly, WBCs were isolated from whole blood via lysis of red blood cells (RBCs) followed by centrifugation. WBCs were resuspended in Phosphate Buffered Saline (PBS) and treated with a proprietary CTC enrichment medium (CEM) that induces cell death in all apoptosis-competent non-malignant (hemato-lymphoid, epithelial, and endothelial) cells, while malignant tumor derived cells (CTCs) survive due to apoptosis resistance. After treatment for 5 days at 37°C, surviving cells and cell clusters are harvested by gentle centrifugation (400 × *g*, 5 min, 4°C) and resuspended in PBS.

Method S4. Immunocytochemistry Profiling of CTCs

Apoptosis reluctant cells enriched from 5 mL of blood were resuspended in 1500 μL 1x Phosphate Buffered Saline (PBS) and 100 μL aliquots of these cells were seeded into 15 wells. Cells were fixed with 4% Paraformaldehyde (15 min), permeabilized with 0.3% Triton-X 100 (5 min), and treated with 3% BSA (blocking, 45 min). Separate aliquots of cells were immunostained (60 min) with the 3 separate Primary (1°) Ab cocktails for multiplexed analysis of the following combination of markers, (a) Anti-CK + Anti-CD45 + Anti-EpCAM, (b) Anti-CK + Anti-CD45 + Anti-CA19.9, (c) Anti-CK, Anti-CD45, Anti-Maspin. Samples for CA19.9 and Maspin were incubated with secondary (2°) anti-mouse Ab (60 min). All antisera were used at dilutions validated for each batch for consistent fluorescence signal of controls. Finally, cells were treated with 4',6-Diamidino-2-phenylindole dihydrochloride (DAPI) for nuclear staining (15 min). Each of the above steps were separated by gentle washes with 1x PBS. All steps were performed at ambient temperature (20°C – 25°C). Run controls included PL45 (positive for CK, EpCAM, CA19.9 and Maspin, negative for CD45) and MOLT3 (positive for CD45, negative for all other markers). Stained samples in multiwell plates

[1] Akolkar D, et al. Circulating ensembles of tumor-associated cells: A redoubtable new systemic hallmark of cancer. Int J Cancer. 2020 Jun 15;146(12):3485-3494. doi: 10.1002/ijc.32815

were evaluated on the CellInsight High Content Screening (HCS) Platform to determine the fluorescence intensity (FI) of each marker as per standard operating procedures (SOPs) based on manufacturer's instructions. Marker status was ascertained via sequential excitation and acquisition of each marker-fluorophore (DAPI → CD45 → CK → CA19.9 / Maspin / EpCAM). All marker positive cells (CK^+, $EpCAM^+$, $CD45^-$; CK^+, $CA19.9^+$, $CD45^-$; CK^+, $Maspin^+$, $CD45^-$) were quality checked to confirm nucleated status (i.e., $DAPI^+$), nuclear-cytoplasmic ratio (i.e., N:C ≥ 0.7) and expected marker localization to nucleus (DAPI), cytoplasm (CK, Maspin, CA19.9) or cell membrane (EpCAM).

WBCs or viable cells isolated from benign or malignant tumors were resuspended in Phosphate Buffered Saline (PBS) and immunustained as above (for **Figure S1**).

Method S5. Marker Expression in Various Cell types

Study: Reference cells (MOLT3, PL45), malignant tumor derived cells (M-TDC), PB-CTC, pooled CTCs from other cancer types (oCTC: Breast, Lung, Head and Neck, Cervix, Prostate and Ovary), WBC from patients with benign pancreaticobiliary conditions (B-WBC) or from healthy donors (HD-WBC) were immunostained to determine expression status of CK, EpCAM, CA19.9 and Maspin.

Findings: Higher expression of CK and EpCAM was seen in PL45, M-TDC, PB-CTCs and oCTCs, while expression of CA19.9 and Maspin was seen only in PL45, M-TDC and PB-CTC (**Figure S1**).

Method S6. Marker Expression in PBCs

Study: FI for CA19.9, Maspin, EpCAM and CK were evaluated in subsets of PB-CTCs enriched from blood samples of patients with pancreatic, gallbladder and bile duct AD, stratified by age, gender or stage of cancer (**Table S1**).

Findings: There were no significant variations in expression any marker due to age-group, gender, primary organ or stage indicating that the test can detect PB-CTCs irrespective of these variables **(Figure S2 – S4**).

Method S7. PB-CTCs in Non-malignant Conditions

Study: To determine the Specificity of the Test to discern PBC from PBB, we evaluated blood samples from 20 individuals who were recently diagnosed with PBB. Samples were processed for CTC enrichment and ICC profiling as described above.
Findings: Among the blood samples from 20 known PBB cases (**Table S2**), PB-CTCs were not detected in any of the samples.

Method S8. Analyte Stability

Study: To determine the Analyte Stability, 4 × 5 mL of blood was collected from 5 known cases of PBC and processed at various time points including within 24 h (baseline), 24 h - 48 h, 48 h - 72 h and 72 h - 96 h after storage at 2°C - 8°C.
Findings: The recovery of marker positive cells at 0h – 24h (baseline) was initially normalized (considered as 100%). Then the recoveries at all other time points were represented as a fraction of the baseline. Based on this approach, ≥85% mean recovery of marker positive cells was observed up to 72 h in all patient samples. The minimal (<15%) reduction in recovery up to 72 h at 2-8°C indicated that samples received within 72 h could be considered pre-analytically equivalent and acceptable.

Method S9. Linearity and Limit of Detection

Study: 168 × 5 mL aliquots of healthy donor blood were divided into 3 sets of 56 aliquots each (1 set per multiplexed marker combination). Aliquots in each set were spiked with (approximately) 1, 3, 5, 10, 20, 40 and 80 PL45 cells (8 replicates each) by serially dilutuing a PL45 master spike (10^5 cells / 5 mL) prepared previously. The study also included 24 × 5 mL aliquots (3 sets × 8 replicates) of healthy donor blood samples which were not spiked. The recoveries of marker positive cells at each spike density were determined to ascertain proportionate (linear) response. The Limit of Detection (LoD) was determined from the replicate data of the 4 lowest level samples, i.e. 1, 3, 5 and 10 cells / 5 mL, as per the method described in CLSI EP17A2.
Findings: The recovery of marker positive cells showed linear characteristics in the evaluated range (1 – 80 cells / 5 mL) with no hook effect. The LoD was determined to be 2 cells / 5 mL for each of the 3 types of marker positive cells.

Method S10. Limit of Blank

Study: The Limit of Blank (LoB) was determined from the 24 × 5 mL unspiked healthy donor blood samples in the Linearity study.
Findings: Since no CA19.9+, Maspin+ or EpCAM+ cells were detected in the unspiked samples (no false positives), the marker-wise and overall LoB was 0 cells / mL. The LoB study indicates the high specificity of the test, i.e., absence (low risk) of false positive findings in absence of analyte (marker positive cells).

Method S11. Interference
Study: The performance characteristics of the Test were evaluated in presence of endogenous (serum markers) and exogenous (non-anticancer drugs) factors as possible interfering agents (**Table S3**). Analytical grade molecules were used to prepare working stock solutions and immediately used for spiking studies. All drugs were evaluated at the reported Peak Plasma Concentrations (C_{Max}), while serum markers were evaluated at concentrations that are considered as elevated. Peripheral blood (72 × 5 mL) from asymptomatic donors who had not taken any medication in the last 14 days was spiked with ~10 PL45 cells each. Each aliquot was used for detection of marker positive cells. Detection of either type of cells in all samples indicated absence of any interference from any of the above drugs.
Findings: The presence of non-anticancer drugs at medically relevant peak plasma concentrations (C_{Max}) or the serum parameters evaluated did not significantly impact the recovery or detection of marker positive cells spiked into blood samples since recovery of marker positive cells was ≥ 80% in presence of any interfering agent (as compared to controls without any interfering agent). The test is expected to remain unaffected in presence of systemic treatment agents (drugs) and elevated serum parameters.

Method S12. Inter-Operator Agreement
Study: Inter-operator (n = 2) agreements were determined using blood samples (10 mL) from a cohort of 75 individuals including 30 PBC cases, 15 PBB cases (**Table S4**) and 30 asymptomatic individuals (no prior diagnosis or current suspicion of cancer). Samples were split into two aliquots of 5 mL each and one aliquot provided to each of two independent operators who were unaware of the clinical status of the samples. Samples were evaluated and reported as ‘C’ (cancer) or ‘B/H’ (benign/healthy). Test

findings were compared with the clinical status to determine (a) concordance of marker findings with clinical status and (b) inter-operator concordance.

Findings: The sample-type wise findings are provided in **Table S5.** The overall agreement (OA) was 96%, positive agreement (PA) was 90% (n = 30 cancers) and negative agreement (NA) was 100% (n = 15 benign conditions and 30 healthy individuals). Neither operator reported marker positive cells in samples from asymptomatic individuals or those with benign conditions. Operator 1 reported all cancer samples as positive while operator 2 reported 3 cancer samples as negative.

SUPPLEMENTARY TABLES

Table S1. Marker Expression Study Cohort: PBC cases.

	Pancreas	Gallbladder	Bile Duct	Overall
**Age Group**				
< 40 years	*10*	*7*	*2*	***19***
41 – 50 years	*10*	*10*	*4*	***24***
51 – 60 years	*10*	*10*	*9*	***29***
61 – 70 years	*10*	*10*	*8*	***28***
> 70 years	*10*	*7*	*7*	***24***
**Gender**				
Female	*20*	*20*	*10*	***50***
Male	*20*	*20*	*10*	***50***
**Stage**				
Stage I	*10*	*5*	*2*	***17***
Stage II	*10*	*8*	*5*	***23***
Stage III	*10*	*7*	*5*	***22***
Stage IV	*10*	*7*	*5*	***22***

Table S2. Marker Expression Study Cohort: Non-malignant Conditions.

Parameter	Value
**Age**	
Median	48 years
Range	17 – 81 years
**Gender**	
Female	10
Male	10
**Diagnosis**	
Benign Bile duct Stricture	1
Cholecystitis	5
Pancreatitis	14

Table S3. Potentially Interfering Substances. The endogenous factors represent the most commonly observed variables during blood pathology work-up. The exogenous factors selected for evaluation of interference represent the most prescribed non-anticancer medications in the US and Europe.

Endogenous Factors	Exogenous Factors
Bilirubin,<br>Cholesterol,<br>Glucose,<br>Haemoglobin,<br>Uric Acid	Levothyroxine,<br>Lisinopril,<br>Atorvastatin,<br>Metformin,<br>Amlodipine,<br>Metoprolol,<br>Omeprazole,<br>Albuterol,<br>Ranitidine,<br>Azithromycin,<br>Paracetamol,<br>Aspirin,<br>Loperamide,<br>Dextromethorphan,<br>Cortisone,<br>Ulipristal Acetate,<br>Sildenafil Citrate,<br>EDTA

Table S4. Inter-Operator Concordance Study: Participant Demographics.

	Pancreas	Gallbladder	Bile Duct	Combined
**Cancers**				
**N =**	12	13	5	30
**Age (years)**				
Median	62	53	46	57
Range	(23 – 79)	(19 – 66)	(36 – 72)	(19 – 79)
**Gender**				
Male	5	4	3	12
Female	7	9	2	18
**Stage**				
Stage I	5	1	1	7
Stage II	1	1	1	3
Stage III	1	2	2	5
Stage IV	5	9	1	15
**Benign conditions**				
**N =**	13	2	-	15
**Age (years)**				
Median	36	62	-	38
Range	(6 – 71)	(62 – 62)	-	(6 – 71)
**Gender**				
Male	7	2	-	9
Female	6	-	-	6
**Healthy (asymptomatic) adults**				
**N =**	-	-	-	30
**Age (years)**				
Median	-	-	-	38
Range	-	-	-	(24 – 56)
**Gender**				
Male	-	-	-	30
Female	-	-	-	-

Table S5. Inter-Operator Concordance Study: Findings

Clinical status			Classification			
			Operator 1		Operator 2	
**Sample Type**	**Type**	**N =**	**C**	**B/H**	**C**	**B/H**
**All Healthy**	**H**	**30**	**-**	**30**	**-**	**30**
**All Benign**	**B**	**15**	**-**	**15**	**-**	**15**
Cholecystitis	*B*	*2*	*-*	*2*	*-*	*2*
Pancreatitis	*B*	*11*	*-*	*11*	*-*	*11*
Pancreas IMT	*B*	*2*	*-*	*2*	*-*	*2*
**All Cancers**	**C**	**30**	**30**	**-**	**27**	**3**
Pancreas AD	*C*	*12*	*12*	*-*	*9*	*3*
Stage 1		*5*	*5*	*-*	*4*	*1*
Stage 2		*1*	*1*	*-*	*-*	*1*
Stage 3		*1*	*1*	*-*	*-*	*1*
Stage 4		*5*	*5*	*-*	*5*	*-*
Gallbladder AD	*C*	*13*	*13*	*-*	*13*	*-*
Stage 1		*1*	*1*	*-*	*1*	*-*
Stage 2		*1*	*1*	*-*	*1*	*-*
Stage 3		*2*	*2*	*-*	*2*	*-*
Stage 4		*9*	*9*	*-*	*9*	*-*
Bile Duct AD	*C*	*5*	*5*	*-*	*5*	*-*
Stage 1		*1*	*1*	*-*	*1*	*-*
Stage 2		*1*	*1*	*-*	*1*	*-*
Stage 3		*2*	*2*	*-*	*2*	*-*
Stage 4		*1*	*1*	*-*	*1*	*-*
B: benign; H: healthy; C: cancer; IMT: Inflammatory myofibroblastic tumor (benign)						

Table S6. Case Control Clinical Study: Inclusion and Exclusion Criteria.

	Cancer Cases	Healthy Adults
**Inclusion**<br>(all)	Adult males and females,<br>Recent diagnosis of PBC,<br>Treatment Naïve	Adult males and females,<br>Aged 40 years and above,<br>No history of cancer diagnosis,<br>No present suspicion of cancer,<br>Normal serum CA19.9,<br>Normal USG (A+P),<br>Asymptomatic.
**Exclusion**<br>(any)	Other cancers,<br>Received anticancer treatments	Prior diagnosis of cancer,<br>Currently suspected of cancer,<br>Elevated serum CA19.9,<br>Suspicious findings on USG

Table S7. Case Control Clinical Study: Participant Demographics. This study included 5 mL blood samples from 188 recently diagnosed and therapy naïve PBC cases (cases) and 172 asymptomatic individuals (controls).

Parameter	Cancer Cases				Healthy Adults
	Pancreas	Gallbladder	Bile Duct	Overall	
**N =**	**117**	**44**	**27**	**188**	**172**
**Age (years)**					
Median	60	55	60	60	49
Range	13 - 83	19 - 74	36 - 81	13 - 83	40 - 82
**Gender**					
Female	48	27	8	83	70
Male	69	17	19	105	102
**Stage**					
Stage I	36	11	3	50	
Stage II	41	11	9	61	
Stage III	20	10	7	37	
Stage IV	20	12	8	40	

Table S8. Case Control Clinical Study: Training Set Findings. The Training Set of 252 samples included 132 PBC cases and 120 asymptomatic individuals. All 120 samples from asymptomatic individuals were negative for PB-CTCs. Among the 132 samples from cancer patients, 126 were positive for PB-CTCs and 6 were negative.

Sample Type	Negative	Positive
**Asymptomatic (n = 120)**	**120 (100.0%)**	**0 (0.0%)**
**All Cancers (n = 132)**	**6 (4.5%)**	**126 (95.5%)**
Stage I (n = 35)	*6 (17.1%)*	*29 (82.9%)*
Stage II (n = 43)	-	*43 (100.0%)*
Stage III (n = 26)	-	*26 (100.0%)*
Stage IV (n = 28)	-	*28 (100.0%)*
Cancer-wise		
**Pancreas (n = 82)**	**4 (4.9%)**	**78 (95.1%)**
Stage I (n = 25)	*4 (16.0%)*	*21 (84.0%)*
Stage II (n = 29)	-	*29 (100.0%)*
Stage III (n = 14)	-	*14 (100.0%)*
Stage IV (n = 14)	-	*14 (100.0%)*
**Gallbladder (n = 31)**	**1 (3.2%)**	**30 (96.8%)**
Stage I (n = 8)	*1 (12.5%)*	*7 (87.5%)*
Stage II (n = 8)	-	*8 (100.0%)*
Stage III (n = 7)	-	*7 (100.0%)*
Stage IV (n = 8)	-	*8 (100.0%)*
**Bile Duct (n = 19)**	**1 (5.3%)**	**18 (94.7%)**
Stage I (n = 2)	*1 (50.0%)*	*1 (50.0%)*
Stage II (n = 6)	-	*6 (100.0%)*
Stage III (n = 5)	-	*5 (100.0%)*
Stage IV (n = 6)	-	*6 (100.0%)*

Table S9. Prospective Clinical Study: Participant Demographics. The prospective clinical study included 88 individuals suspected of PBC, who were advised a biopsy for diagnosis.

**Parameter**	**Post HPE Status of all samples**				
	Cancers				**Benign**
	Pancreas	***Gallbladder***	***Bile Duct***	***Combined***	
**N =**	23	19	7	49	39
**Age (years)**					
Median	58	45	60	56	38
Range	33 – 70	31 – 62	29 - 78	29 - 78	21 - 81
**Gender**					
Female	5	13	6	24	21
Male	18	6	1	25	18
**Cancer Stage#**					
Stage I	12	9	-	21	
Stage II	2	2	1	5	
Stage III	4	2	4	10	
Stage IV	5	6	2	13	
**Benign Condition#**					
Bile Duct Stricture					2
Cholecystitis					4
Cholelithiasis					5
Pancreatic BIMT*					2
Pancreatitis					24
Heterotypic Pancreas					1
Cystic Fibrosis of Pancreas					1

**Benign Inflammatory myofibroblastic tumor.*

Table S10. Prospective Clinical Study: Findings. Based on observed marker expression profile in the 88 samples, 50 were classified Positive and 38 were classified Negative.

Sample Type	Negative	Positive
**Benign (n = 39)**	**36 (92.3%)**	**3 (7.7%)**
**All Cancers (n = 49)**	**2 (4.1%)**	**47 (95.9%)**
Stage I (n = 21)	*2 (9.5%)*	*19 (90.5%)*
Stage II (n = 5)	-	*5 (100.0%)*
Stage III (n = 10)	-	*10 (100.0%)*
Stage IV (n = 13)	-	*13 (100.0%)*
Cancer-wise		
**Pancreas (n = 23)**	**1 (4.3%)**	**22 (95.7%)**
Stage I (n = 12)	*1 (8.3%)*	*11 (91.7%)*
Stage II (n = 2)	-	*2 (100.0%)*
Stage III (n = 4)	-	*4 (100.0%)*
Stage IV (n = 5)	-	*5 (100.0%)*
**Gallbladder (n = 19)**	**1 (5.3%)**	**18 (94.7%)**
Stage I (n = 9)	*1 (11.1%)*	*8 (88.9%)*
Stage II (n = 2)	-	*2 (100.0%)*
Stage III (n = 2)	-	*2 (100.0%)*
Stage IV (n = 6)	-	*6 (100.0%)*
**Bile Duct (n = 7)**	**-**	**7 (100.0%)**
Stage I (n = 0)	-	-
Stage II (n = 1)	-	*1 (100.0%)*
Stage III (n = 4)	-	*4 (100.0%)*
Stage IV (n = 2)	-	*2 (100.0%)*

SUPPLEMENTARY FIGURES

Figure S1. Marker Expression in Various Cell types. The expression (FI: fluorescence intensity; as relative fluorescence units, RFU) of CK, EpCAM, CA19.9 and Maspin were evaluated on various cell types.

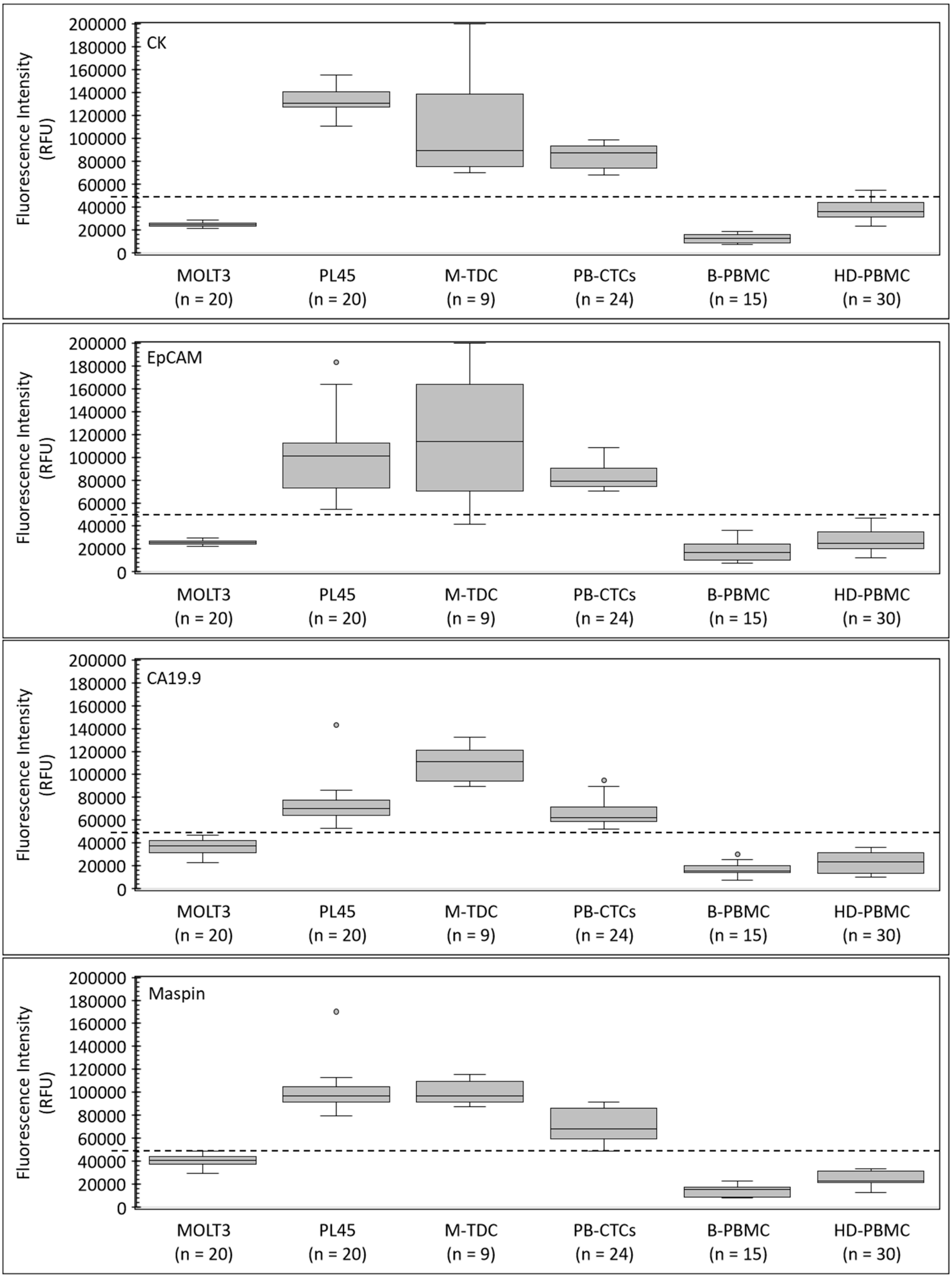

Figure S2. PBC Stage and Marker Expression. The expression (FI: fluorescence intensity; as relative fluorescence units, RFU) of CK, EpCAM, CA19.9 and Maspin in (P) Pancreas, (GB) Gallbladder, (BD) Bile duct, cancers stratified by (St) Stage.

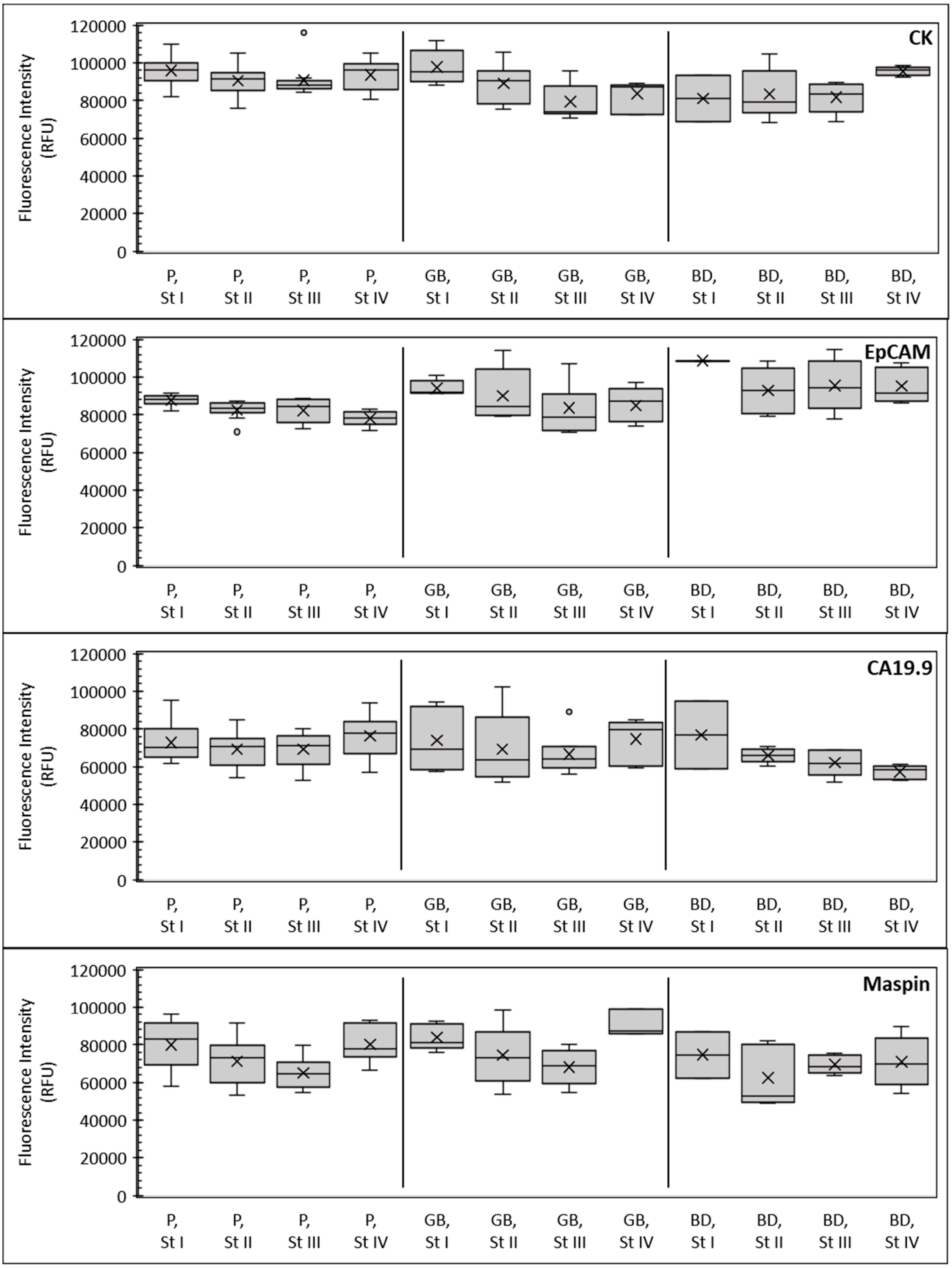

Figure S3. Patient Age and Marker Expression. The expression (FI: fluorescence intensity; as relative fluorescence units, RFU) of CK, EpCAM, CA19.9 and Maspin in (P) Pancreas, (GB) Gallbladder, (BD) Bile duct cancers stratified by patient age (years).

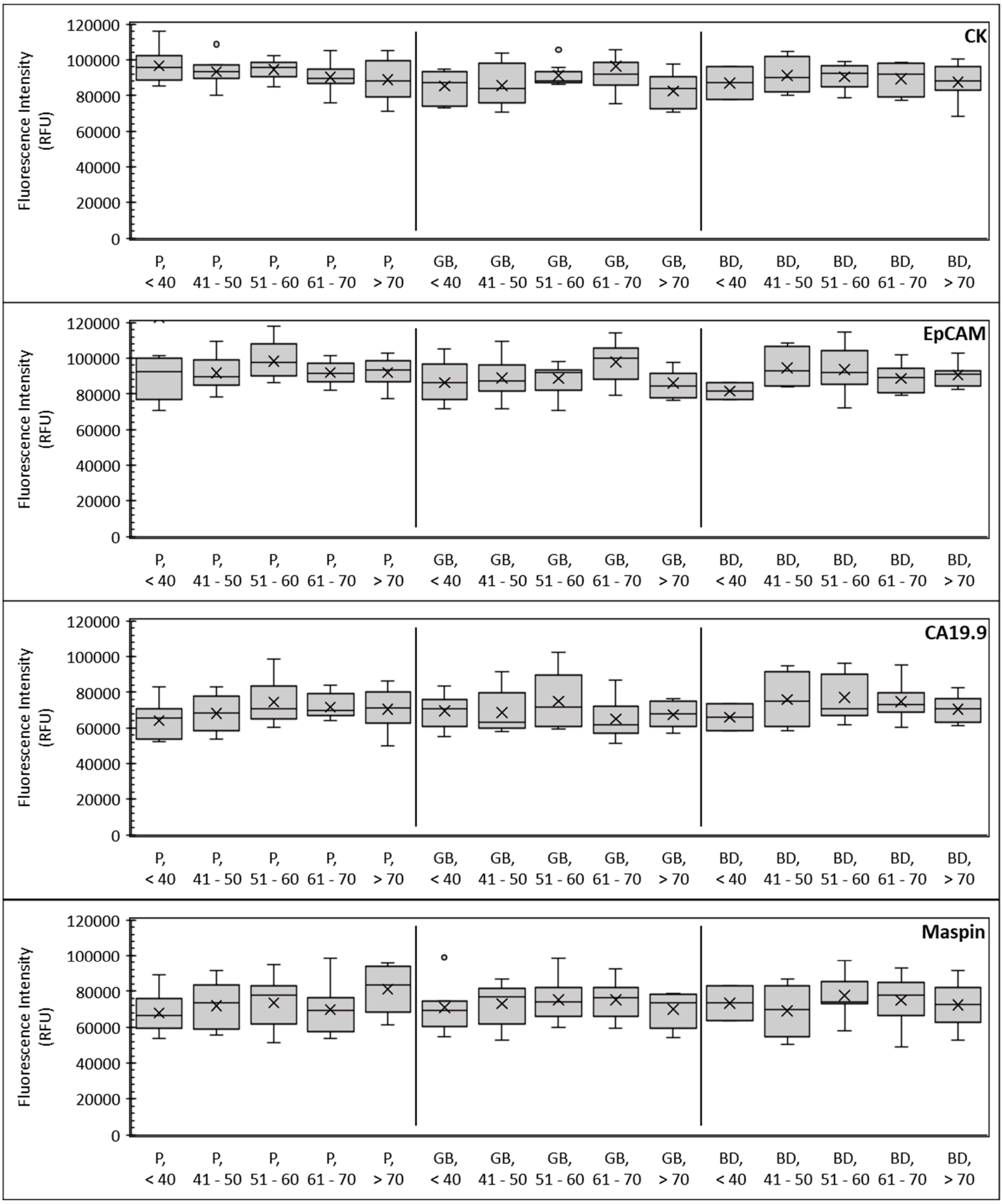

Figure S4. Patient Gender and Marker Expression. The expression (FI: fluorescence intensity; as relative fluorescence units, RFU) of CK, EpCAM, CA19.9 and Maspin in (P) Pancreas, (GB) Gallbladder, (BD) Bile duct cancers in (F) Female and (M) Male.

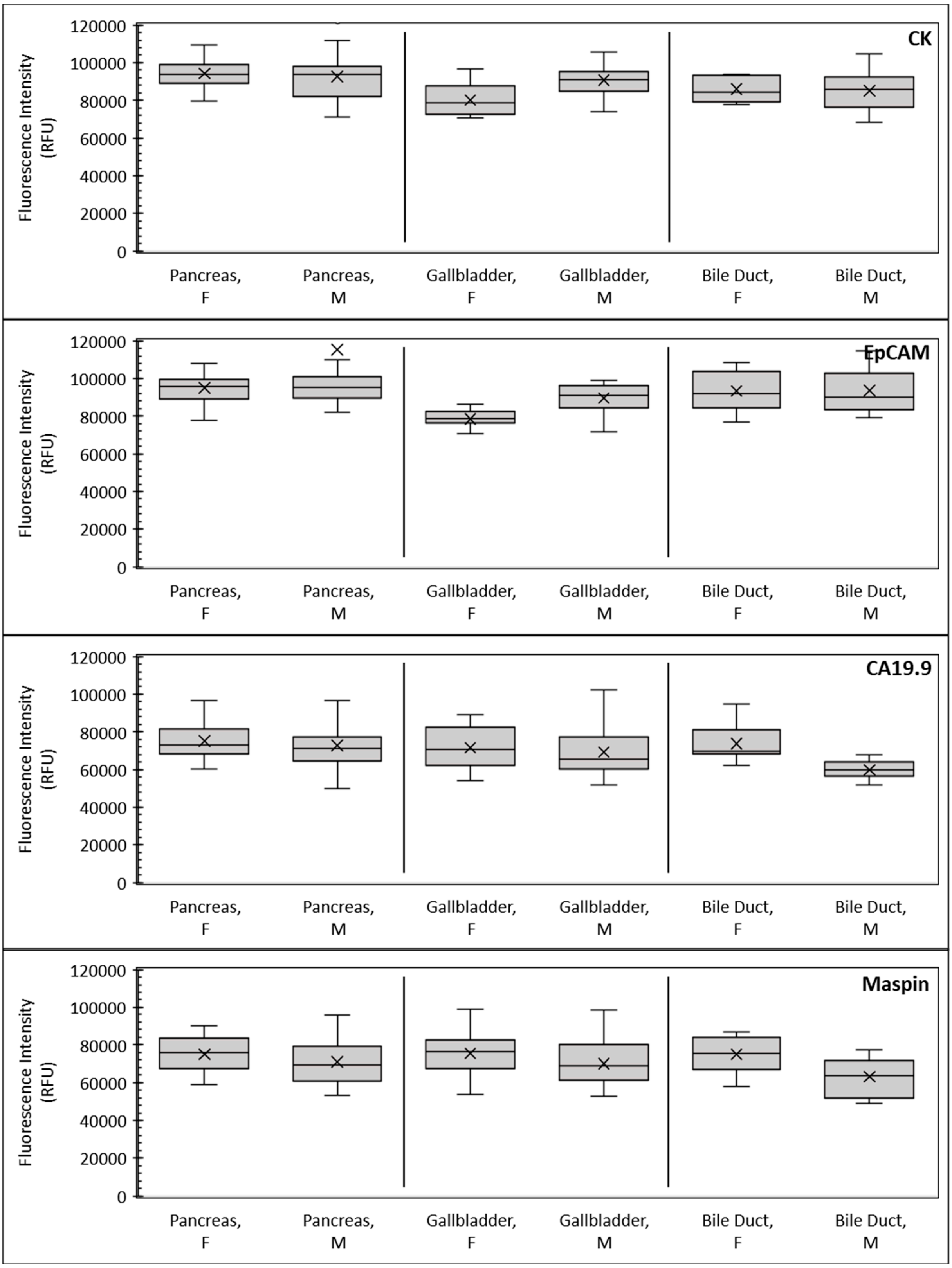

Supplement: Supplementary file 1 [file cancers-16-01400-s001.zip › cancers-2930803-supplementary.pdf]
